# Avian Influenza Virus (H5N1) Mortality Surveillance

**DOI:** 10.3201/eid1407.080161

**Published:** 2008-07

**Authors:** Nicholas Komar, Björn Olsen

**Affiliations:** * Centers for Disease Control and Prevention, Fort Collins, Colorado, USA; † Uppsala University, Uppsala, Sweden

**Keywords:** Avian mortality, surveillance, influenza, bird, Europe, H5N1, HPAI, letter

**To the Editor:** The highly pathogenic strain of avian influenza virus subtype H5N1 presents a major challenge to global public health systems. Currently, influenza (H5N1) infection is a zoonosis with a 60% case-fatality rate for affected persons over 3 continents; the virus could mutate to become directly transmissible among humans (*1*). This potential for pandemic transmission must be reduced through early detection of transmission foci, followed by rapid implementation of control measures (*2*). In the following analysis, we demonstrate that single carcasses of birds, mostly found by members of the public, were the primary indicators for avian influenza virus activity in Sweden and Denmark in 2006.

Influenza virus (H5N1) is amplified by commercial and backyard poultry and free-ranging birds. Whether captive birds (e.g., poultry) or wild birds are responsible for the spread of the virus remains a matter of debate (*3*). Initial spread from Southeast Asia before 2005 was likely the result of transport of infected poultry because the spread was not easily explained by natural bird movements (*4*,*5*). However, its spread to Western Europe in late 2005 could be explained by weather-induced migration of waterfowl after a freeze in Eastern Europe (*6*,*7*). Since spreading to Sweden and Denmark in early 2006, the virus has been detected there in dead birds of numerous species (Table). Detections in carcasses of primarily free-ranging birds have become the principal means of tracking spread of the virus in Europe.

**Table:** Bird species testing positive for highly pathogenic avian influenza virus subtype H5N1, Sweden and Denmark, 2006*

Avian order and species	Scientific name	No. carcasses positive	
		Sweden	Denmark
Podicepidiformes (great crested grebe)	*Podiceps cristatus*	0	1
Anseriformes			
Mute swan	*Cygnus olor*	2	4
Whooper swan	*Cygnus cygnus*	0	3
Greylag goose	*Anser anser*	0	1
Goose spp	*Anser spp.*	1	0
Muscovy duck	*Cairina moschata*	0	2
Mallard	*Anas platyrhynchos*	1	0
Greater scaup	*Aythya marila*	3	0
Tufted duck	*Aythya fuligula*	25	26
Common merganser	*Mergus merganser*	2	0
Smew	*Mergus albellus*	1	0
Falconiformes			
Common buzzard	*Buteo buteo*	1	6
Rough-legged hawk	*Buteo lagopus*	0	1
Peregrine falcon	*Falco peregrinus*	0	1
Galliformes			
Common peafowl	*Pavo cristatus*	0	1
Domestic chicken	*Gallus gallus*	0	1
Charadriiformes (herring gull)	*Larus argentatus*	1	0
Strigiformes (eagle owl)	*Bubo bubo*	2	0
Passeriformes (Eurasian magpie)	*Pica pica*	0	1
All birds		39	48

*Sources (*6*,*7*).

To better understand how avian mortality surveillance could be refined for monitoring the spread of influenza virus (H5N1), we analyzed the weekly official reports of such detections in Sweden and Denmark in 2006 (*8*). Virus surveillance in both countries includes both active cloacal swabbing of free-ranging wild birds and passive collection of tracheal swabs from bird carcasses. For the analysis, all carcasses of a single species collected on 1 day within a single locality constituted 1 record. For each record, we evaluated whether the carcass(es) were reported by a member of the public versus a civil servant, the number of carcasses tested, and the number of positive detections.

Our analysis evaluated 44 records; a total of 70 birds, of 14 species, tested positive for the virus in 22 localities of Sweden and Denmark. Almost all of these records (n>40, 91%) referred to dead birds found by members of the public rather than civil servants. A smaller portion than expected were Anseriformes (i.e., ducks, geese, or swans; n = 32, 73%). Other orders of birds represented were Falconiformes (hawks, falcons; n = 8, 18%), Strigiformes (owls; n = 2, 5%), Podicepidiformes (grebes; n = 1, 2%), and Charadriiformes (gulls, shorebirds; n = 1, 2%). In addition, birds of other orders tested positive in Denmark but were excluded from the analysis for lack of supporting data. Most (75%) of the records referred to singleton carcasses; the remaining 25% represented multiple detections, ranging from 2 to 9 individual birds of a single species. A majority (73%) of influenza virus (H5N1)–positive localities hosted solely singleton carcasses, whereas the other 27% hosted multiple dead birds. No virus activity was detected through active free-ranging bird surveillance, even though 9,260 live birds were captured and sampled during 2006 in Sweden and Denmark.

The pattern of virus activity observed in Sweden and Denmark was unexpected. Rather than die-offs of large numbers of waterfowl during winter when they congregate, small numbers (mainly singleton birds) were affected late in winter, just before spring migration. During the spring breeding season, less transmission was observed. The predictive power of detecting the virus in free-ranging migratory birds for forecasting poultry outbreaks or human disease remains undetermined. Some of these birds may have been infected in areas remote from the site of detection. However, several of the affected birds in this report were either resident nonmigratory species (eagle owl, Eurasian magpie) or captive domesticated species (muscovy, peafowl, chicken), which indicates local transmission. Health authorities will be better prepared to prospectively minimize transmission in new regions with early warning provided by singleton carcass surveillance.

Surveillance results from Sweden and Denmark highlight the importance of public participation in avian mortality surveillance for influenza virus (H5N1); the preponderance of detections from singleton carcasses; and the broad spectrum of affected species, particularly raptors. A raptor was the index case in Denmark (*7*). Current surveillance efforts in regions free from the virus favor investigation of significant death events of waterfowl and active sampling of healthy waterfowl as the means for early detection (e.g., *9*). Many national surveillance programs are heavily influenced by the influenza virus (H5N1) outbreak in 2005 at Qinghai Lake in China, where hundreds of geese, gulls, and cormorants died during the breeding season (*10*). However, large die-offs may be anomalous or restricted to communal breeding sites of waterfowl where juvenile birds amplify and spread the virus within the breeding colony. Testing of public-reported singleton carcasses provides a more sensitive and robust means of early detection of this virus.

## References

[1] Peiris JS, de Jong MD, Guan Y. Avian influenza virus (H5N1): a threat to human health. Clin Microbiol Rev. 2007;20:243–67. 10.1128/CMR.00037-06 17428885 PMC1865597

[2] Mumford E, Bishop J, Hendrickx S, Embarek PB, Perdue M. Avian influenza H5N1: risks at the human–-animal interface. Food Nutr Bull. 2007;28:S357–63. 17658082 10.1177/15648265070282S215

[3] Feare CJ. The role of wild birds in the spread of HPAI H5N1. Avian Dis. 2007;51(Suppl):440–7. 10.1637/7575-040106R1.1 17494603

[4] Olsen B, Munster VJ, Wallensten A, Waldenström J, Osterhaus AD, Fouchier RA. Global patterns of influenza A virus in wild birds. Science. 2006;312:384–8. 10.1126/science.1122438 16627734

[5] Gauthier-Clerc M, Lebarbenchon C, Thomas F. Recent expansion of highly pathogenic avian influenza H5N1: a critical review. Ibis. 2007;149:202–14. 10.1111/j.1474-919X.2007.00699.x

[6] Kilpatrick AM, Chmura AA, Gibbons DW, Fleischer RC, Marra PP, Daszak P. Predicting the global spread of H5N1 avian influenza. Proc Natl Acad Sci U S A. 2006;103:19368–73. 10.1073/pnas.0609227103 17158217 PMC1748232

[7] Bragstad K, Jørgensen PH, Handberg K, Hammer AS, Kabell S, Fomsgaard A, First introduction of highly pathogenic H5N1 avian influenza A viruses in wild and domestic birds in Denmark, Northern Europe. Virol J. 2007;4:43. 10.1186/1743-422X-4-43 17498292 PMC1876802

[8] Swedish Board of Agriculture. Weekly reports Fågelinfluensa [in Swedish] [cited 2008 June 9]. Available from http://www.sjv.se

[9] Cattoli G, Terregino C, Guberti V, De Nardi R, Drago A, Salviato A, Influenza virus surveillance in wild birds in Italy: results of laboratory investigations in 2003–2005. Avian Dis. 2007;51(Suppl):414–6. 10.1637/7562-033106R.1 17494596

[10] Liu J, Xiao H, Lei F, Zhu Q, Qin K, Zhang XW, Highly pathogenic H5N1 influenza virus infection in migratory birds. Science. 2005;309:1206. 10.1126/science.1115273 16000410

